# Lifestyle intervention for type 2 diabetes patients – trial protocol of The Copenhagen Type 2 Diabetes Rehabilitation Project

**DOI:** 10.1186/1471-2458-9-166

**Published:** 2009-05-29

**Authors:** Eva S Vadstrup, Anne Frølich, Hans Perrild, Eva Borg, Michael Røder

**Affiliations:** 1 Department of Endocrinology and Gastroenterology, Bispebjerg University Hospital, Copenhagen, Denmark; 2 Department of Integrated Healthcare, Bispebjerg University Hospital, Copenhagen, Denmark; 3 Health Care Centre Oesterbro, Copenhagen, Denmark; 4 Department of Cardiology and Endocrinology, Hillerød University Hospital, Hillerød, Denmark

## Abstract

**Background:**

Current guidelines recommend education, physical activity and changes in diet for type 2 diabetes patients, yet the composition and organization of non-pharmacological care are still controversial. Therefore, it is very important that programmes aiming to improve non-pharmacological treatment of type 2 diabetes are developed and evaluated. The Copenhagen Type 2 Diabetes Rehabilitation Project aims to evaluate the effectiveness of a new group-based lifestyle rehabilitation programme in a Health Care Centre in primary care.

**Methods/Design:**

The group-based diabetes rehabilitation programme consists of empowerment-based education, supervised exercise and dietary intervention. The effectiveness of this multi-disciplinary intervention is compared with conventional individual counselling in a Diabetes Outpatient Clinic and evaluated in a prospective and randomized controlled trial. During the recruitment period of 18 months 180 type 2 diabetes patients will be randomized to the intervention group and the control group. Effects on glycaemic control, quality of life, self-rated diabetes symptoms, body composition, blood pressure, lipids, insulin resistance, beta-cell function and physical fitness will be examined after 6, 12 and 24 months.

**Discussion:**

The Copenhagen Type 2 Diabetes Rehabilitation Project evaluates a multi-disciplinary non-pharmacological intervention programme in a primary care setting and provides important information about how to organize non-pharmacological care for type 2 diabetes patients.

**Trail Registration:**

**ClinicalTrials.gov **registration number: NCT00284609.

## Background

Type 2 diabetes mellitus (T2DM) is a chronic disease with severe late complications and high mortality. The increasing prevalence of T2DM is mainly due to reduced physical activity and consumption of unhealthy food and larger portion sizes in genetic susceptible individuals. Lifestyle intervention can prevent development of T2DM in subjects with impaired glucose tolerance [1,2]. To improve metabolism and reduce the risk of late complications of T2DM, permanent changes in lifestyle and lifelong multi-pharmacological treatment are needed [3].

Group-based lifestyle intervention programmes for T2DM patients, including patient education or supervised exercise, have been evaluated in several randomized controlled trials. Education programmes alone improves glycaemic control in some studies [4,5], but not in all [6]. Group-based diabetes education seems to have a better effect on glycaemic control than individual education [7-10]. However the studies and interventions included in these meta-analyses are very heterogeneous.

Isolated aerobic exercise [11], resistance training [12], and the combination of these[13] resulted in better glycaemic outcomes compared with the control groups. Exercise in groups (exercise period ranging from 8 weeks to 12 month) lowered HbA1c approximately 0.6 percentage points [14,15].

Dietary advice is an accepted cornerstone of treatment for T2DM, but no quality data on the efficacy of diet intervention per se on glycaemic control, reduction in body weight, development of diabetic complications or quality of life exists for the treatment of T2DM [16].

It is still an open question whether group-based or individual lifestyle intervention programmes gives the best glycaemic control, whether exercise interventions have a long term effect and finally whether lifestyle changes can improve long term results of T2DM treatment.

A new health care centre is established at Oesterbro in eastern Copenhagen as a result of a local collaboration project between the City of Copenhagen, Bispebjerg University Hospital and general practitioners [17]. The health care centre is planned to be responsible for lifestyle rehabilitation of patients with one or more of four selected chronic conditions – type 2 diabetes, chronic obstructive pulmonary disease, chronic heart failure and older patients with balance problems. A new rehabilitation programme combines empowerment-based education, exercise training and dietary advice on a group-based level as one multi-disciplinary intervention.

The aim of the current study is to compare the metabolic, physiological and psychological effects of this new group-based multi-disciplinary lifestyle rehabilitation programme for T2DM patients in a primary care setting with an individual counselling programme in a diabetes outpatient clinic. We hypothesize that patients participating in the group-based rehabilitation programme including supervised exercise will improve their glycaemic control, self-rated diabetes symptoms and quality of life significantly more in the short and long term than patients receiving conventional individual advice on changes in lifestyle.

## Methods/design

### Patient recruitment and randomization

**Figure 1:**
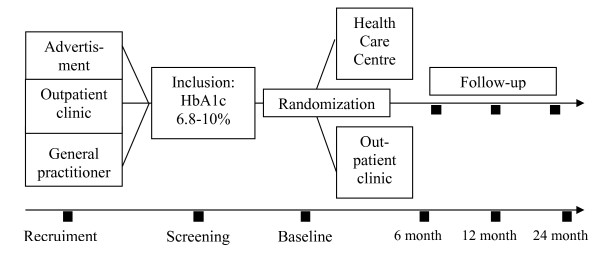
**Flow-chart of events in the study**. From recruiting patients through randomization and follow-up.

**Figure 2:**
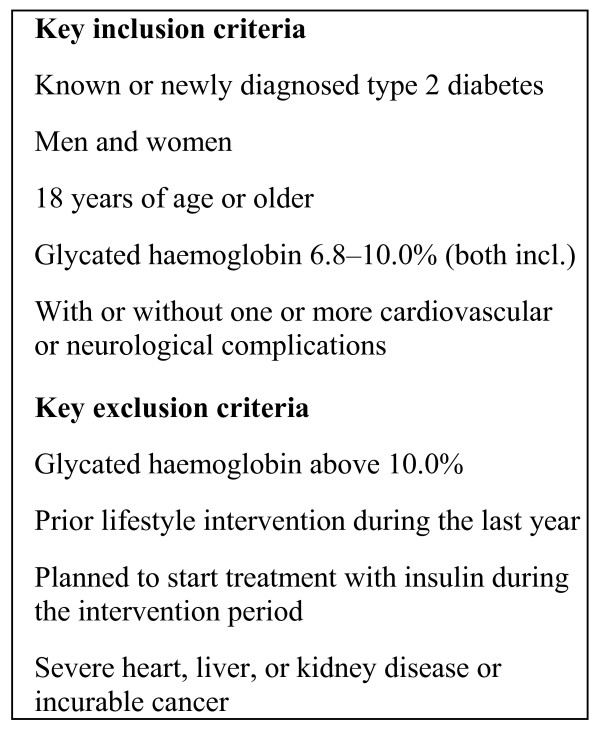
**Key inclusion and exclusion criteria**.

We plan to recruit patients through advertisements in local newspapers, pharmacies and from the outpatient clinic at Bispebjerg University Hospital, and by letters and e-mails to general practitioners inviting them to refer patients to the study (Figure 1). Key inclusion and exclusion criteria are shown in Figure 2. Diagnosis of T2DM is defined according to the criteria of WHO [18]. Patients willing to participate will be screened after written informed consent is obtained, and those fulfilling the inclusion criteria will be randomised within three weeks, stratified by gender and age (18–54 years and ≥ 55 years). A person not participating in the study creates a randomization list, and the randomization is done at the baseline visit using consecutively numbered sealed envelopes. Patients will be randomised to a group-based rehabilitation programme (intervention group) at the Health Care Centre Oesterbro or to an individual counselling programme (control group) in the Diabetes Outpatient Clinic, Bispebjerg University Hospital.

#### Intervention group

**Figure 3:**
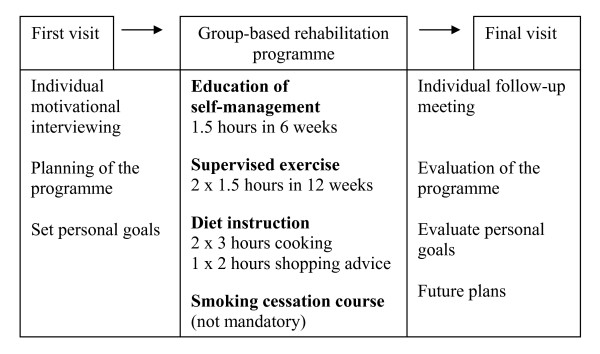
**The group-based rehabilitation programme outline**.

Lifestyle rehabilitation at Health Care Centre Oesterbro consists of a multi-disciplinary intervention including three programmes (Figure 3).

**The education programme **consists of one weekly group session of 90 minutes for 6 weeks, limited to 8 patients per group. A nurse, a physiotherapist, a dietician and a podiatrist run the sessions. The education programme is originally developed at the diabetes outpatient clinic, Bispebjerg University Hospital, and focuses on achievement of self-selected goals, psychosocial adaptation, enhanced control and problem solving strategies [19]. The programme is modified and adjusted to a multi-disciplinary education team, and the contents of the sessions include information on the pathophysiology of diabetes; the role inheritance and environment; education in self blood-glucose monitoring; the importance of physical activity, healthy diet, weight loss, medication, and smoking cessation; the late complications of T2DM; hypertension and cardiovascular disease. The podiatrist advises patients about foot care and examines the patients' feet. The empowerment approach to diabetes education involves patient collaboration and seeks to increase the patients' knowledge and self-awareness, which enables them to take charge of their own diabetes self-management [20].**The diet instruction programme **consists of two 3-hours group-based cooking courses and 1 session in a local supermarket [21]. Patients' spouses are encouraged to join the cooking courses.

**The physical exercise programme **is supervised and is 90 minutes in duration and sessions take place twice a week for 12 weeks. The sessions are group-based, but a physiotherapist tailors an individual exercise programme for each patient including both aerobic and resistance exercise [22]. After initial measurement of blood-glucose levels, the patients start the warm-up exercise for 20 minutes. This is followed by basic exercise training for 15 minutes, followed by aerobic exercise on a bicycle ergometer for 12 minutes, resistance training (seated leg press, chest press, pull down and leg extension) for 25 minutes, stretch for 10 minutes, followed by a final blood-glucose measurement. The patients are advised to perform 30 minutes of physical activity on at least three additional days per week according to international guidelines [23]. In addition patients are offered an optional **smoking cessation course **if needed.

Before patients enter the programme they participate in an individual motivational interview with a nurse or a physiotherapist. The personnel are educated and continuously supervised in the use of the motivational interviewing technique by a specialized psychologist [24]. The patients set personal goals, and these goals are evaluated after the intervention programme at a final consultation and 1 and 3 months later by telephone follow-up contacts.

The teams from the health care centre and from the diabetes outpatient clinic meet on a regular basis to share knowledge and to ensure a common understanding of care and agreement on the components of the programmes in the two organizations.

### Control group

**Figure 4:**
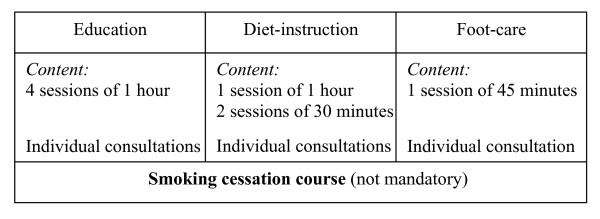
**Individual outpatient counselling programme outline**.

On the basis of the same empowerment approach the lifestyle intervention at the Diabetes Out-patient clinic at Bispebjerg Hospital includes four monthly 1-hour sessions of individual counselling with a diabetes nurse specialist [19,20] (Figure 4). The nurse is educated in the use of the motivational interviewing technique and has a bachelor degree in education. Using storytelling patients are given information about diabetes in general, medication, risk factors and late complications of T2DM. They receive guidance in self-monitoring of blood-glucoses and on how to increase their level of physical activity.

The programme also includes three individual counselling sessions with a dietician, who is also educated in the use of the motivational interviewing technique[24]. The patients' set personal goals and the dietician develops in cooperation with the patient, a diet-schedule based on biochemical, anthropometrical and medical records including patients' motivation and attitudes. The schedule and goals are evaluated at two 30-minute follow-up visits.

The patients receive 45 minutes guidance and instructions about foot-care from a podiatrist. Finally patients are offered an optional smoking cessation course if required.

### Pharmacological treatment

Both intervention programmes are non-pharmacological. The endocrinologist or the general practitioner treating and monitoring the patients prior to the study continue as the patients' physician (disease manager) during and after the intervention. Recent guidelines on pharmacological treatment are followed [25]. The study does not include changes in principles for the pharmacological treatment. Patients are asked to report any alternation in their medication and these will be quantified by the study investigator and used in the analyses as a covariate.

### Outcome measures and data collection

The primary outcome measure is glycosylated haemoglobin (HbA1c). Secondary outcome measures are self-rated quality of life and diabetes symptoms. Explanatory outcome measures include blood pressure, lipid profile, body weight, waist circumference, insulin resistance and beta-cell function estimates, and physical fitness. All outcome measures are collected at baseline and at follow-up visits after 6, 12 and 24 months.

At every visit, biochemical, anthropometrical, physiological and physical values are obtained. The same investigator measures the anthropometrical and the physiological outcomes throughout the study. Physiotherapists at the health care centre and at the outpatient clinic complete the physical tests. The patients fill in two self-administered questionnaires on quality of life and diabetes related symptoms at each visit.

### Anthropometrical measurements

Weight (kg) is measured without shoes in indoor clothing and the same equipment will be used throughout the study. Height (cm) without shoes is only measured at baseline.

Waist circumference (cm) is measured at a level midway between the lower rib margin and iliac crest with the subjects standing without clothes on and with relaxed breathing.

### Physiological measurements

Electrocardiogram (ECG) is recorded at baseline. Blood pressure is measured on both arms are done using an aneroid manometer with the patient in a sitting position after 10–15 minutes of relaxed conversation. The mean of 3 consecutive measurements is used for each arm. Patients' target blood pressures are at the recommended goal of 130/80 mmHg defined according to the European guidelines [26].

Biothesiometry of the pulp on the first toe on both feet's is used for quantitative assessment of peripheral neuropathy. The patients will be tested by increasing the amplitude from zero and then the patients will indicate when they feel the vibration. The cut-off point for ulcer risk is > 25 volts [27].

### Biochemical measurements

Blood and urine samples are taken in the morning after an overnight fast. Plasma glucose, lipids, C Reactive Protein, creatinine and urine albumin/creatinine ratio are analysed at the Department of Clinical Biochemistry, Bispebjerg University Hospital. Haemoglobin A1c is measured on venous whole blood samples in K2-EDTA tubes at the Department of Clinical Biochemistry, Steno Diabetes Centre [28]. Blood samples for serum C-peptide, insulin and proinsulin are centrifuged for 10 min at 3000 rpm, at 20°C immediately after sampling, and serum is isolated and frozen before analysis at the Steno Diabetes Centre. Blood samples for later analyses will be centrifuged within 60 minutes of sampling and stored frozen at -20°C. The target for haemoglobin A1c is below 6.5% according to international guidelines [27].

The Homeostasis Model Assessment (HOMA) is used to estimate insulin resistance and beta-cell function [29]. HOMA-IR is computed as follows: fasting insulin (μU/ml) × fasting glucose (mmol/L)/22.5. HOMA-B is calculated using the following formula: 20 × fasting insulin (μU/ml)/fasting glucose (mmol/L) – 3.5.

Microalbuminuria is defined as a urine Albumin:Creatinine Ratio (ACR) ≥ 2.5 – 25 mg/mmol in men and ≥ 3.5 – 25 mg/mmol in women [30]. Treatment targets for blood lipids are values of fasting total cholesterol below 4.5 mmol/L and fasting LDL cholesterol below 2.5 mmol/L [31].

### Physical measurements

Patients above 60 years of age perform a 6 minutes walk test in an indoor unobstructed corridor. They are instructed by a physical therapist to walk the 30 meter from one end to the other at their own pace, as many times as possible, in the permitted time. After 6 minutes, the total distance walked (in meter) is measured. The 6 minute walk test is a useful measure of functional capacity, targeted at older people and people with moderate-to-severe physical impairment and has well known reference equations [32,33].

For patients below 60 years of age physical fitness is measured by using a submaximal cycle ergometer aerobic fitness test (Astrand-Rhyming Cycle Ergometer Test). Patients cycle on an ergometer at a constant workload for 6 minutes. Heart rate is measured every minute, and based on the steady state heart rate, VO_2 _max (correlation to VO_2 _max approximately 0.85–0.9) is determined from a nomogram [34,35].

All participants perform a strength test that involves the determination of the maximum weight that can be lifted 5 repetitive times by arms and legs while maintaining proper form (5RM test) [36]. Self reported daily physical activity (average hours per week in the last 6 months) is divided into: 1) Transportation exercise: walking and bicycling and 2) Training exercise: jogging, training in a fitness centre, swimming, bicycling, etc[36].

### Quality of life questionnaires

Short Form Health Survey (SF-36) is a multi-purpose, short-form health survey with 36 questions. It yields an 8-scale profile of functional health and well-being scores as well as psychometrically-based physical and mental health summary measures and a preference-based health utility index. The SF-36 has proven to be useful in surveys of general and specific populations, comparing the relative burden of diseases, and has been used to differentiate the health benefits produced by a wide range of different treatments [37]. A higher score on SF-36 indicates an improvement in Quality of Life.

Diabetes Symptom Checklist – Revised (DSC-R) is a self-report questionnaire of 34 questions grouped into 8 symptom subscales measuring the occurrence and perceived burden of diabetes-related symptoms [38]. It has been described to be valid, reliable and responsive to change and to be the only scale that appears to evaluate physical functioning in T2DM patients in a broad, comprehensive manner [39]. A lower score on DSC-R indicates an improvement in Quality of Life.

### Sample size considerations and statistical methods

The size of the study population is based on the primary outcome measure HbA1c and is estimated on the basis of the results of two meta-analyses relating to education and exercises in T2DM patients [8,14]. According to power calculation, an absolute difference of 0.7% in HbA1c between the groups (power 0.9, significance level 0.05) may be detected with 80 patients in each group. This calculation is based on a standard deviation of the HbA1c-value of 1.3 from larger population studies [40].

Neither patients nor study personnel are blinded to treatment assignment. The study statistician carrying out the data analysis on the primary and secondary outcomes will be blinded and will not have any contact with the patients.

Descriptive statistics (means ± SD, or median and interquartile ranges, as appropriate) will be used to describe the study sample with regards to baseline characteristics. The analysis will be performed according to the intention-to-treat principles using the statistical software SAS 9.1. Comparisons of primary and secondary outcomes between the two groups will be analyzed by values after the intervention at the 6 months follow-up visit, and 12 and 24 months after baseline, using appropriate parametric tests for variables fulfilling the normal distribution criteria or appropriate non-parametric tests for variables not fulfilling the normal distribution criteria. Sensitivity analyses will be carried out with adjustment for possible confounders. When relevant the change in outcomes from baseline to 6, 12 and 24 months follow-up will be assessed. The proportion of patients reaching the recommended goals for glycated haemoglobin, blood pressure and lipids will be compared in a sub-analysis. Statistical significance is set at P < 0.05.

### Ethics

The study will be conducted according to the principles of the Helsinki declaration. The Danish National Committee on Biomedical Research Ethics and the Danish Data Protection Agency have approved the study protocol.

## Discussion

This prospective, controlled and randomized open label study tests the hypothesis that a new multi-disciplinary group-based lifestyle intervention programme for T2DM patients in a primary health care setting results in better short- and long-term glycaemic outcomes than the traditional best practice for individual lifestyle counselling in a diabetes outpatient clinic setting. The major difference between the two interventions is that the new rehabilitation programme includes a supervised exercise programme, whereas the control group receives advice regarding physical activity. The content and the principles of empowering patients are the same in the two interventions, but the education programme in the health care centre is group-based.

The evidence of the beneficial effects of exercise on diabetes symptoms, physical fitness and quality of life in T2DM is strong [41], and previous group-based non-pharmacological lifestyle studies show improvement in glycaemic control and weight loss compared to individual counselling [9]. Most lifestyle intervention studies have been focused mainly on education, including advice on physical activity and diet, or on exercise training and/or diet. Only a few randomized controlled trials have investigated interventions incorporating several elements of non-pharmacological treatment of T2DM including patient education, supervised exercise and dietary advice on a group-based level [42-45]. However, the number of patients, follow-up time and effects on glycaemic control were very heterogeneous in these studies. It is very important in T2DM management programmes to find ways to increase the impact of non-pharmacological treatment in T2DM patients. Our study may add further evidence to this.

The lifestyle rehabilitation programme is multi-disciplinary including group-based education, dietary advice and exercise, and it is not possible to extract the individual effects of each component. The interventions are non-pharmacological, but the patients are urged to continue their medication throughout the study since the clinical effects of a multi-pharmacological treatment on late diabetic complications are highly significant [46]. Decisions on changes in medication are left to the discretion of the patients' physicians, who are not part of this study.

Disease management programmes tailored to the Danish health care system are currently developed for T2DM and other chronic conditions. Provision of rehabilitation programmes for T2DM patients in a community-based setting, and health care centres, are a possible future part of the organization of prevention and chronic care in primary care, and it is therefore essential to provide evidence for the patient-related effects. The evidence generated from The Copenhagen Type 2 Diabetes Rehabilitation Project may be helpful in the decision making of providers to choose the most suitable lifestyle programme for their T2DM patients.

## Competing interests

The authors declare that they have no competing interests.

## Authors' contributions

ESV drafted the manuscript. ESV, AF, HP, EB and MR participated in the design of the study and provided input into the main ideas of this paper. ESV, AF, HP, EB and MR obtained funding for the project. ESV, AF, HP, EB and MR read, commented, and approved the final version of the manuscript.

## Pre-publication history

The pre-publication history for this paper can be accessed here:


